# Identification of receptor-type protein tyrosine phosphatase μ as a new marker for osteocytes

**DOI:** 10.1007/s00418-015-1319-1

**Published:** 2015-04-08

**Authors:** Karien E. de Rooij, Martijn van der Velde, Edwin de Wilt, Martine M. L. Deckers, Martineke Bezemer, Jan H. Waarsing, Ivo Que, Alan. B. Chan, Eric L. Kaijzel, Clemens W. G. M. Löwik

**Affiliations:** Experimental Molecular Imaging, Department of Radiology, Leiden University Medical Center, Albinusdreef 2, PO Box 9600, 2300 RC Leiden, The Netherlands; Percuros BV, Enschede, The Netherlands; Department of Orthopaedics, Erasmus MC, Rotterdam, The Netherlands

**Keywords:** Osteocytes, RPTPμ, Bone, Micro-CT, Animal model

## Abstract

Osteocytes are the predominant cells in bone, where they form a cellular network and display important functions in bone homeostasis, phosphate metabolism and mechanical transduction. Several proteins strongly expressed by osteocytes are involved in these processes, e.g., sclerostin, DMP-1, PHEX, FGF23 and MEPE, while others are upregulated during differentiation of osteoblasts into osteocytes, e.g., osteocalcin and E11. The receptor-type protein tyrosine phosphatase µ (RPTPμ) has been described to be expressed in cells which display a cellular network, e.g., endothelial and neuronal cells, and is implied in mechanotransduction. In a capillary outgrowth assay using metatarsals derived from RPTPμ-knock-out/LacZ knock-in mice, we observed that the capillary structures grown out of the metatarsals were stained blue, as expected. Surprisingly, cells within the metatarsal bone tissue were positive for LacZ activity as well, indicating that RPTPμ is also expressed by osteocytes. Subsequent histochemical analysis showed that within bone, RPTPμ is expressed exclusively in early-stage osteocytes. Analysis of bone marrow cell cultures revealed that osteocytes are present in the nodules and an enzymatic assay enabled the quantification of the amount of osteocytes. No apparent bone phenotype was observed when tibiae of RPTPμ-knock-out/LacZ knock-in mice were analyzed by μCT at several time points during aging, although a significant reduction in cortical bone was observed in RPTPμ-knock-out/LacZ knock-in mice at 20 weeks. Changes in trabecular bone were more subtle. Our data show that RPTPμ is a new marker for osteocytes.

## Introduction

Osteocytes are the most abundant cell type in bone and are involved in several processes in bone homeostasis and metabolism as extensively reviewed by Bonewald (2011). When osteoblasts become embedded in the newly synthesized bone matrix, they develop dendritic processes, change shape and thus differentiate into osteocytes. During this process, osteocalcin levels are elevated (Mikuni-Takagaki et al. 1995) and E11 is expressed (Wetterwald et al. 1996; Schulze et al. 1999) which may play a role in osteocyte morphology (Zhang et al. 2006). Osteocytes are involved in phosphate metabolism and matrix mineralization through the proteins PHEX, MEPE, FGF23, BSP and DMP-1, which are all strongly expressed by osteocytes (reviewed in Atkins and Findlay 2012). The osteocyte-derived protein sclerostin plays a key role in regulation of bone formation by modulating BMP and Wnt signaling (van Bezooijen et al. 2004; Ten Dijke et al. 2008; Moester et al. 2010). The position of the osteocytes within the mineralized matrix and the network of cellular processes make them very well positioned to act as “the nerve cells of the bone.” They have been shown to be sensitive to fluid flow shear stress (Klein-Nulend et al. 1995). Tatsumi et al. (2007) have reported that targeted ablation of osteocytes in mice induced bone loss with deterioration of bone architecture. However, upon mechanical unloading, these mice did not show bone loss compared with control mice. In addition, the absence of connexin 43 in osteoblasts/osteocytes also protected against mechanical unloading, while increasing the effect of mechanical loading (Lloyd et al. 2013; Bivi et al. 2013), implying an important role for osteocytes in mechanical induced bone homeostasis.

The receptor-like protein tyrosine phosphatase µ (RPTPµ) belongs to the MAM (*m*eprin/*A*5/RPTP*μ*) containing subfamily of transmembrane protein tyrosine phosphatases. RPTPµ is expressed in some types of neuronal cells, lung epithelium, cardiac muscle cells and distinct endothelial cells (Koop et al. 2003). An important common feature of these cell types is the importance of cell–cell interactions. Strikingly, some of these cells also form a cellular network. RPTPµ is involved in cell–cell interactions (Brady-Kalnay and Tonks 1994; Gebbink et al. 1995; Zondag et al. 1995) and may play a role in the formation or maintenance of the cellular network. Accordingly, little RPTPµ expression is found in fenestrated types of endothelium, as present in the liver or the spleen (Bianchi et al. 1999).

RPTPµ-knock-out/LacZ knock-in mice, in which the expression of the LacZ gene is controlled by the RPTPµ promoter, allow for the analysis of RPTPµ expression in vivo in various tissues (Koop et al. 2003). These mice were shown to have decreased flow-induced dilatation in mesenteric arteries, implying a role for RPTPµ in mechanotransduction (Koop et al. 2005). To analyze the expression of RPTPμ in endothelial cells in an angiogenesis assay, we have performed capillary outgrowth assays using metatarsals derived from RPTPµ-knock-out/LacZ knock-in mice. To our surprise, not only the outgrowing capillaries were positive for LacZ, but also osteocytes within the metatarsal bone tissue showed LacZ expression. Here, we report that, in bone tissue, RPTPµ is expressed exclusively in the osteocytes and therefore is a new marker for osteocytes.

## Materials and methods

### Mice

Homozygous RPTPµ-knock-out/LacZ knock-in mice were obtained from Prof. W.H. Moolenaar (The Dutch Cancer Institute, Amsterdam, The Netherlands). These mice were generated by replacing exon 1 of the RPTPµ gene from the start codon to the splice donor site by the LacZ gene, as described by Koop et al. (2003). Wild-type FVB mice were obtained from Harlan (Horst, The Netherlands). Homozygous RPTPµ-knock-out/LacZ knock-in were crossbred with wild-type FVB mice to generate heterozygous RPTPµ-knock-out/LacZ knock-in mice. Subsequently, these heterozygous RPTPµ-knock-out/LacZ knock-in mice were used to breed wild-type and knock-out littermates for μCT analysis of bone architecture.

Mice were kept in a controlled 12-h/12-h light/dark cycle and had unlimited access to food and water. All animal experiments were performed with approval of the Ethical Committee for animal experiments of the LUMC.

### Immunohistochemistry and β-galactosidase staining

RPTPμ-knock-out/LacZ knock-in mice were killed either by decapitation (5-day-old neonatal mice) or by cervical dislocation (adult mice). Long bones and calvariae were prepared and fixed in LacZ fixative (0.1 mM NaH_2_PO_4_(H_2_O)/2 mM MgCl_2_/5 mM EGTA/0.26 % glutaraldehyde) for 30 min. After washing the bones in LacZ washing buffer (0.1 mM NaH_2_PO_4_(H_2_O)/2 mM MgCl_2_/0.2 % Nonidet P 40/0.1 % deoxycholate) three times for 10 min, they were stained for β-galactosidase activity in LacZ staining solution (LacZ washing buffer supplemented with 1 mg/ml X-gal, 5 mM K_3_Fe(CN)_6_ and 5 mM K_4_Fe(CN)_6_(3H_2_O)) overnight at room temperature. The long bones were postfixed in 3.7 % formaldehyde in PBS overnight at 4 °C, decalcified in 15 % EDTA/0.5 % paraformaldehyde for 2 weeks and embedded in paraffin. Sections (5 µm) were counterstained using the staining procedure for connective tissue according to Von Weichert-Giesson. Images were acquired using a color CCD camera mounted on a Nikon Eclipse 610 microscope. The calvariae were postfixed as described, washed twice in PBS and photographed directly using a Contax 167MT camera mounted on a Zeiss microscope.

Cultures of metatarsals or bone marrow stromal cells were washed two times in PBS, fixed in LacZ fixative for 3 min, washed in LacZ washing buffer three times for 5 min and stained for β-galactosidase activity in LacZ staining solution overnight at room temperature. Following postfixation in 3.7 % formaldehyde in PBS for 10 min, the cultures were washed twice with PBS and photographed.

PECAM-1 staining of metatarsal cultures was performed as described (Deckers et al. 2001). Briefly, the cultures were fixed in zinc macrodex formalin fixative and incubated with ER-MP12 for 16 h at 4 °C. After incubation with a biotinylated secondary antibody, the signal was visualized using the AEC chromogen.

### Capillary outgrowth assay

Capillary outgrowth assays were performed essentially as described (Deckers et al. 2001). In brief, 17-day-old fetuses were isolated from pregnant RPTPμ-knock-out/LacZ knock-in mice, and metatarsals were dissected. Metatarsals were allowed to attach for 72 h in 175 µl minimal essential medium alpha (α-MEM; Gibco, Carlsbad, CA, USA)/penicillin/streptomycin (Gibco)/10 % (v/v) heat-inactivated fetal calf serum (FCS; Greiner Bio-One, Kemsmünster, Austria). After the attachment phase, medium was replaced with 250 µl fresh culture medium. Following 7 more days of culture, outgrowth of capillaries was visualized by staining for PECAM-1 or β-galactosidase activity as described above.

#### Bone marrow cell cultures

RPTPμ-knock-out/LacZ knock-in mice of 8 weeks old were killed by cervical dislocation. Both femurs and tibiae were isolated, the ends were removed, and the bone marrow was obtained by flushing with 5 ml 10 % FCS in PBS. For one differentiation assay, cells of 2–3 mice were pooled, seeded at a density of 1.5 × 10^6^ cells/well in 12-well plates and cultured in phenol red-free α-MEM (Gibco) supplemented with 10 % (v/v) heat-inactivated FCS, penicillin/streptomycin, and 50 µg/ml ascorbic acid (BDH Prolabo, VWR International, Radnor, PA, USA). The bone marrow cells were cultured for 21 days, and medium was refreshed every 3–4 days. From day 11 of culture onward, the cultures were either supplemented with 10 mM β-glycerol phosphate (Sigma-Aldrich, St. Louis, MO, USA) alone or stimulated with BMP-4 (50 ng/ml; R&D systems, Minneapolis, MN, USA), BMP-6 (100 ng/ml; R&D systems) or Noggin (250 ng/µl; R&D systems) as well. At day 21, the culture medium was withdrawn and the cell layers were processed for β-galactosidase activity determination either by staining with X-gal or by an enzymatic assay using *o*-nitro-phenyl-β-D-galactopyranoside (ONPG; Sigma-Aldrich). Parallel cultures were washed twice with PBS, fixed in 3.7 % formaldehyde in PBS for 5 min and stained with 2 % alizarin red S solution (Sigma-Aldrich).

#### Enzymatic assay for the quantification of β-galactosidase activity in bone marrow cultures

Cell cultures were washed twice with PBS and lysed in 500 µl ALP lysis buffer (10 mM glycine/0.1 mM MgCl_2_/10 µM ZnCl_2_/0.1 % Triton X-100) overnight at 4 °C. Of this lysate, 100 µl was added to 150 µl of LacZ assay buffer (0.1 M K_2_HPO_4_/0.1 % Triton X-100/0.02 M Na_2_HPO_4_/2.2 mM KCl/0.2 mM MgSO_4_/7 mM 2-mercaptoethanol) containing 1 mg/ml *o*-nitro-phenyl-β-D-galactopyranoside (ONPG) and incubated for 3 h at 37 °C. The amount of *o*-nitro-phenol formed was determined by measuring the absorption of the assay solution at 405 nm using a ThermoMax microplate reader (Molecular Devices, Sunnyvale, CA, USA). The β-galactosidase activity was corrected for the amount of DNA in the cultures. To release the DNA from the cell debris, the remaining cell lysate was incubated with an equal volume of SCC buffer containing 100 µg/ml proteinase K (Invitrogen, Carlsbad, CA, USA) for 40 h at 56 °C. DNA content was measured using Hoechst 33258 (ICN Biomedicals, Inc., Irvine, CA, USA) and calibrated against a DNA standard (0.5–10 µg/ml herring sperm DNA; Invitrogen).

#### Reverse-transcribed polymerase chain reaction (RT-PCR)

Total RNA was isolated from bone marrow cell cultures at five time points (days 7, 10, 14, 17 and 21) during differentiation with Trizol LS reagent (Invitrogen). Subsequently, cDNA was synthesized using random primers and M-MLV reverse transcriptase (Promega, Madison, WI, USA) according to the manufacturer’s instructions. Expression of alkaline phosphatase, osteocalcin, LacZ and Sost was determined by quantitative RT-PCR using the QuantiTect SYBR Green PCR kit (Qiagen, Venlo, The Netherlands) with a CFX96 Touch Real-Time PCR Detection System (BioRad, Hercules, CA, USA). Primers (Eurogentec, Seraing, Belgium) were designed using Primer Blast; sequences are given in Table 1. Measurements were performed in triplicate and analyzed using the 2^−ΔΔCt^ method with β2-microglobulin as internal control (Pfaffl 2001).

**Table 1:** Primers used in quantitative RT-PCR for the analysis of gene expression during osteogenic differentiation of bone marrow-derived stem cells

Gene	Forward primer	Reverse primer
*β* *2-Microglobulin*	5′-TGACCGGCTTGTATGCTATC-3′	5′-CAGTGTGAGCCAGGATATAG-3′
*Alkaline phosphatase*	5′-ACACCACAACACGGGCGAGG-3′	5′-TGCCCTCGTTGGCCTTCACG-3′
*Osteocalcin*	5′ -AGAGACAAGTCCCACACAGCAGC-3′	5′ -TGAAGGCTTTGTCAGACTCAGGGC-3′
*LacZ*	5′-CTACGTCTGAACGTCGAAAACCCG-3′	5′-GTAGCGGTCGCACAGCGTGTACCAC-3′
*Sost*	5′-TCCTCCTGAGAACAACCAGAC-3′	5′-TGTCAGGAAGCGGGTGTAGTG-3′

#### Micro-computed tomography

The left tibiae of 8-, 20-, 32-, 44- and 56-week-old RPTPμ-knock-out/LacZ knock-in mice and wild-type littermates (*n* = 5–9 per genotype/age group) were prepared for micro-computed tomography (μCT) by fixing with 3.7 % formaldehyde in PBS for 24 h at 4 °C and stored in 70 % alcohol. They were subsequently scanned using the SkyScan 1076 µCT scanner (SkyScan, Kontich, Belgium) with a source voltage of 40 kV and a current set to 250 µA, using a step size of 0.8° over a trajectory of 180°. Images were made with a voxel size of 9 µm and a frame averaging of 3 to reduce noise. Beam hardening was reduced using a 1-mm aluminum filter. Reconstructions were made using NRecon software (version 1.6.2.0; SkyScan) with a ring artifact correction of 5 and a beam hardening correction set to 20 %. The proximal tibia (0.5–1.5 mm from the growth plate) was chosen as the region of interest to study trabecular and cortical bone. To distinguish calcified tissue from non-calcified tissue, the reconstructed grayscale images were segmented by an automated algorithm using local thresholds (Waarsing et al. 2004). The trabeculae and cortex were separated using PrStackBot-New (software developed by the Orthopedic Research Laboratory, Erasmus MC). The following three-dimensional (3D) bone morphometric parameters were determined using the freely available software package 3D-Calculator (http://www.erasmusmc.nl/47460/386156/Downloads): cortical volume (Ct.V; mm^3^), cortical thickness (Ct.Th; µm), endocortical volume (Ec.V; mm^3^), structure model index (SMI), trabecular separation (Tb.Sp; µm), trabecular volume (Tb.V; mm^3^), trabecular thickness (Tb.Th; µm) trabecular number (Tb.N; mm^−1^) and trabecular connectivity (Conn.). The SkyScan CT-Analyzer software was used to determine the following two-dimensional (2D) parameters: cortical area (Ct.Ar; mm^2^), mean polar moment of inertia (MMI(pol); mm^4^), cortical perimeter (Ct.Pm; mm) and endocortical perimeter (Ec.Pm; mm). Trabecular bone volume fraction (BV/TV; %) was calculated by dividing trabecular volume by endocortical volume, while trabecular connectivity density (Conn.D; mm^−3^) was derived by dividing connectivity by endocortical volume.

### Statistical analysis

Values represent mean ± SEM. Statistical analysis of the normally distributed data was performed with Graphpad Prism 5 software (La Jolla, CA, USA) using one-way analysis of variance (ANOVA), followed by the post hoc Bonferroni test. For statistical analysis of µCT data, a Student’s *t* test was performed. Results were considered significant at *p* < 0.05.

## Results

### RPTPµ expression in angiogenesis of metatarsal explants

**Fig. 1:**
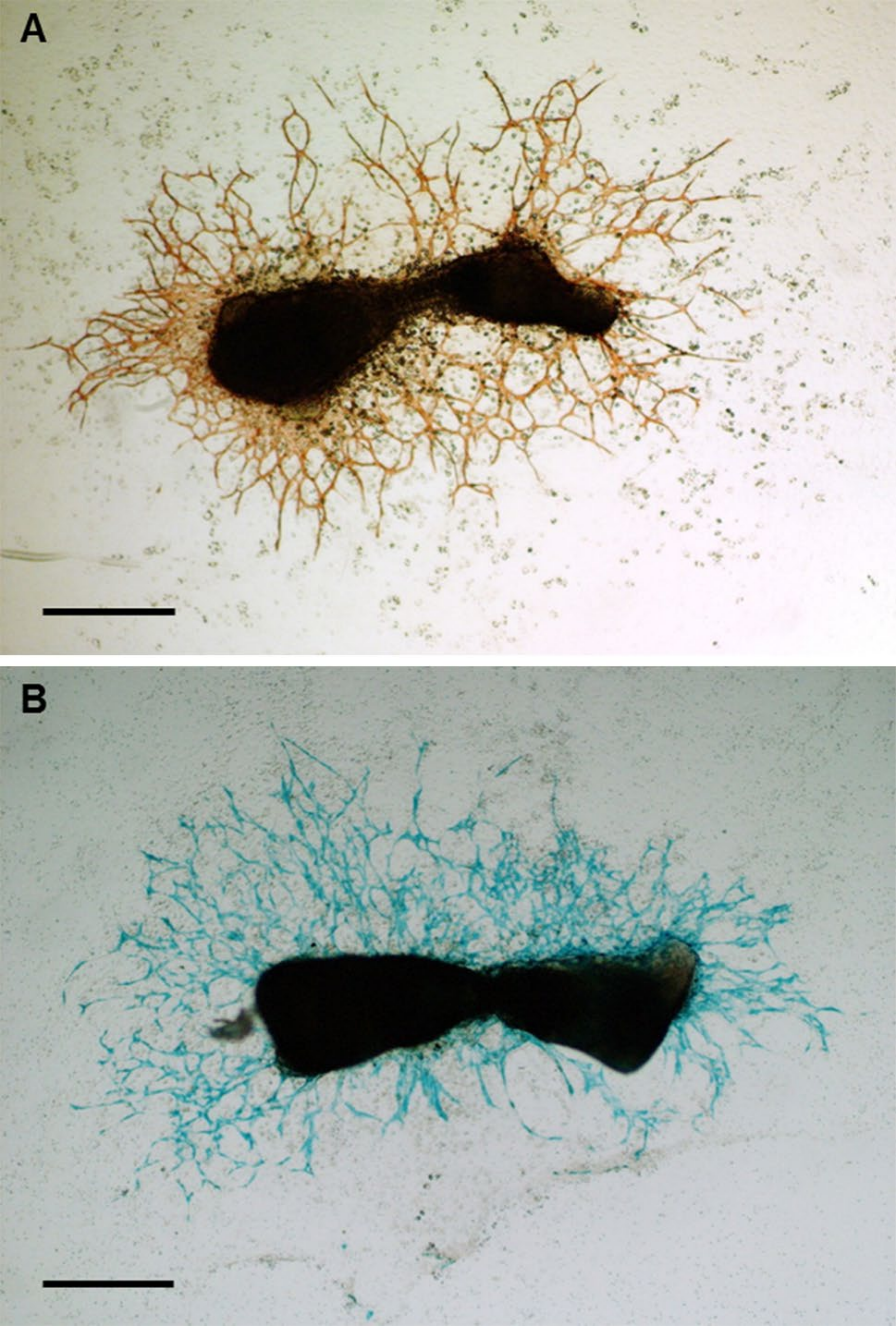
β-Galactosidase activity in capillary outgrowth of metatarsals. Cultures of metatarsals of 17-day-old RPTPμ-knock-out/LacZ knock-in fetuses were stained for the endothelial cell marker PECAM (CD31) (**a**) and LacZ (**b**). The capillary outgrowth shows a clear blue staining, illustrating the expression of RPTPµ in these cells. *Bars* represent 200 µm

**Fig. 2:**
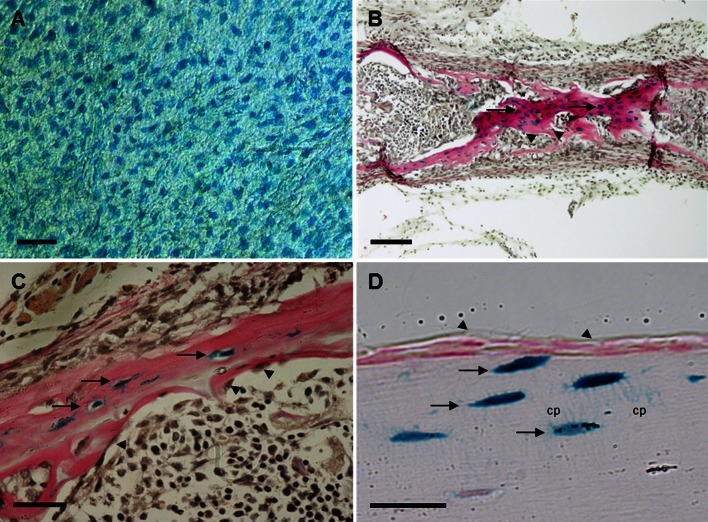
Expression of RPTPµ in bone. Calvariae (**a**), metatarsals (**b**) and tibiae (**c**) of 5-day-old neonatal and tibiae (**d**) of adult RPTPμ-knock-out/LacZ knock-in mice were stained for β-galactosidase activity using X-gal. Within the calvariae, the cellular network of osteocytes is stained *blue*. In neonatal metatarsals and tibiae, the osteocytes (*arrows*) show a deep *blue color*, while the osteoblasts (*arrowheads*) are not stained. The adult tibiae also show that the *blue* staining representing RPTPµ expression is only present in osteocytes (*arrows*), while lining cells (*arrowheads*) are negative. The cellular processes (*cp*) are clearly visible. *Bars*: **a** 50 µm; **b** 100 µm; **c**, **d** 25 µm

To analyze the expression of RPTPµ in the capillary outgrowth of our angiogenesis assay, metatarsals from 17-day-old RPTPμ-knock-out/LacZ knock-in fetuses were dissected, cultured for 10 days and stained for the endothelial cell marker PECAM-1 (CD31) and β-galactosidase activity. During the culture period, a feeder layer of fibroblast-like cells had grown from the metatarsals on which PECAM-1-positive tube-like structures were formed (Fig. 1a). When stained for β-galactosidase activity, the capillary structures showed a deep blue color, demonstrating the expression of the RPTPµ gene in these endothelial cells (Fig. 1b). In addition, within the bone of the metatarsal, the osteocytes were stained blue (see Fig. 2b).

### Expression of RPTPµ within bone

To examine the expression of RPTPµ within bone, calvariae, metatarsals and tibiae of 5-day-old neonatal and tibiae of adult RPTPμ-knock-out/LacZ knock-in mice were isolated and analyzed for β-galactosidase activity by staining with X-gal. Within the neonatal calvariae, the osteocytes were stained for β-galactosidase activity and the cellular network was clearly visible (Fig. 2a). Blood vessels within sutures were also stained (data not shown). Histological sections of neonatal metatarsals and tibiae revealed that within the bone, the osteocytes exhibited β-galactosidase activity, while osteoblasts did not (Fig. 2b, c). In osteoclasts, no β-galactosidase activity was present (not shown). Adult tibiae showed the same expression pattern of RPTPµ. Only osteocytes displayed β-galactosidase activity in contrast to lining cells (Fig. 2d). The blue staining was present throughout the cells, and the cellular processes could be easily distinguished.

### Osteocytes are generated in vitro

**Fig. 3:**
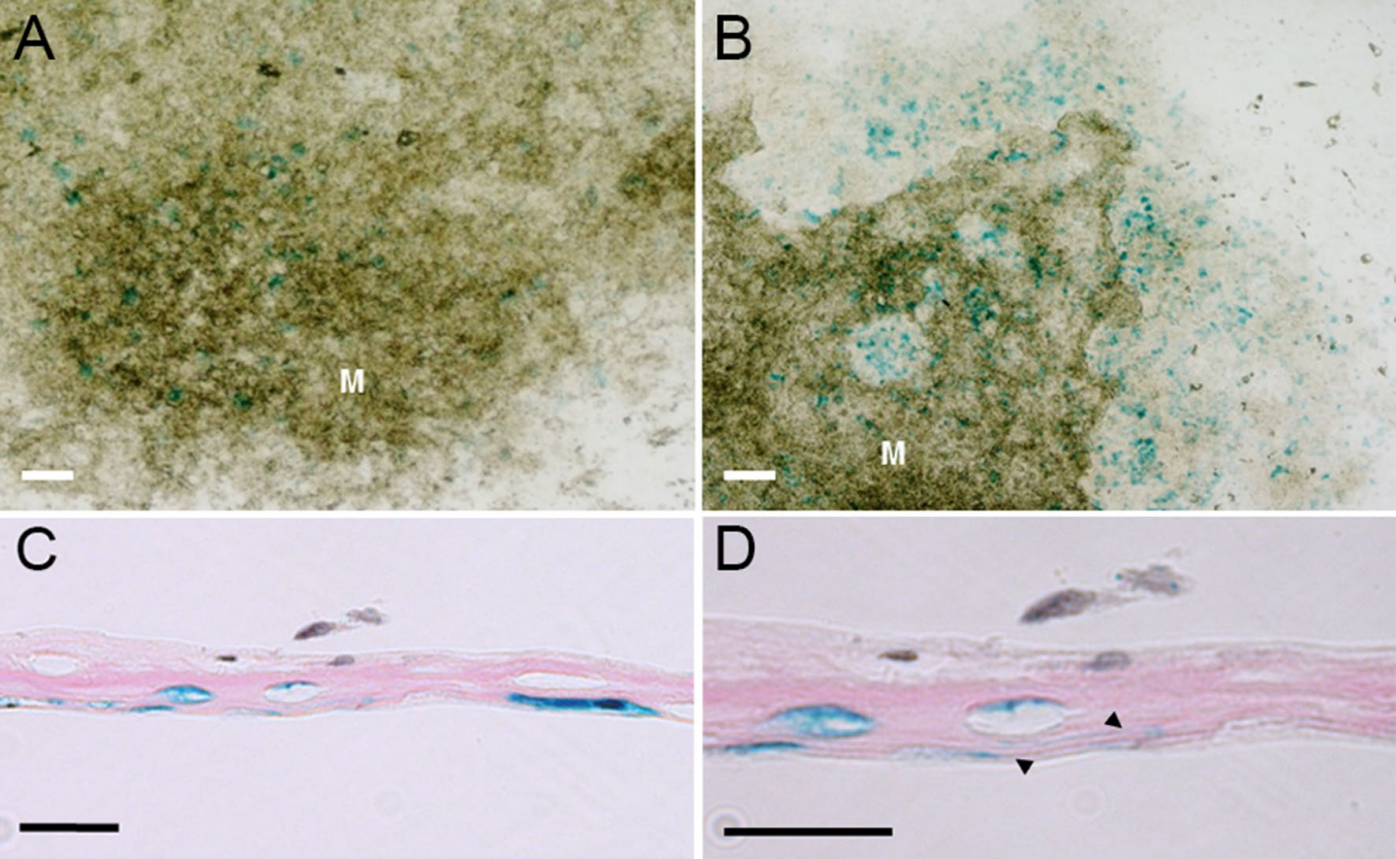
Osteocytes can be generated in vitro. Bone marrow stromal cells (MSC) of RPTPμ-knock-out/LacZ knock-in mice were cultured for 21 days under osteogenic conditions and stained for β-galactosidase activity using X-gal. Within the cultures, *blue*-stained cells can be observed (**a**). The number of *blue*-stained cells is markedly increased when BMPs were added to the medium (**b**). Close observation shows that within the mineralized (M) nodules osteocytes are present, although the number of *blue*-stained cells is higher in the non-mineralized areas of the nodules. Histological analysis of the MSC cultures shows that only cells that are embedded within matrix show β-galactosidase activity (**c**). Some of these cells show cellular processes, which is very characteristic for osteocytes, which can be observed in a larger magnification of the same area (*arrowheads* **d**). *Bars* **a**, **b** 250 µm; **c**, **d** 25 µm

Under osteogenic culture conditions, murine bone marrow stromal cells (MSCs) are able to form nodules in culture. However, it is not known whether within these nodules osteoblasts differentiate into osteocytes. To examine the presence of osteocytes within these nodules, MSCs from RPTPμ-knock-out/LacZ knock-in mice were cultured under osteogenic conditions for 21 days with or without the presence of BMPs. After staining for β-galactosidase activity, positive cells were observed within the cultures (Fig. 3a). Addition of BMPs to the culture medium increased the number of blue-stained cells (Fig. 3b). Close observation showed blue-stained cells within the calcified nodules, but the majority of the blue-stained cells were observed next to the calcified areas. Sections of the cell layers of the differentiated MSC cultures stained with X-gal demonstrated that only cells embedded in matrix show LacZ expression (Fig. 3c). At larger magnification, some of these blue-stained cells showed cellular processes, a characteristic of osteocytes in vivo (Fig. 3d).

### BMPs stimulate osteocyte formation in vitro

**Fig. 4:**
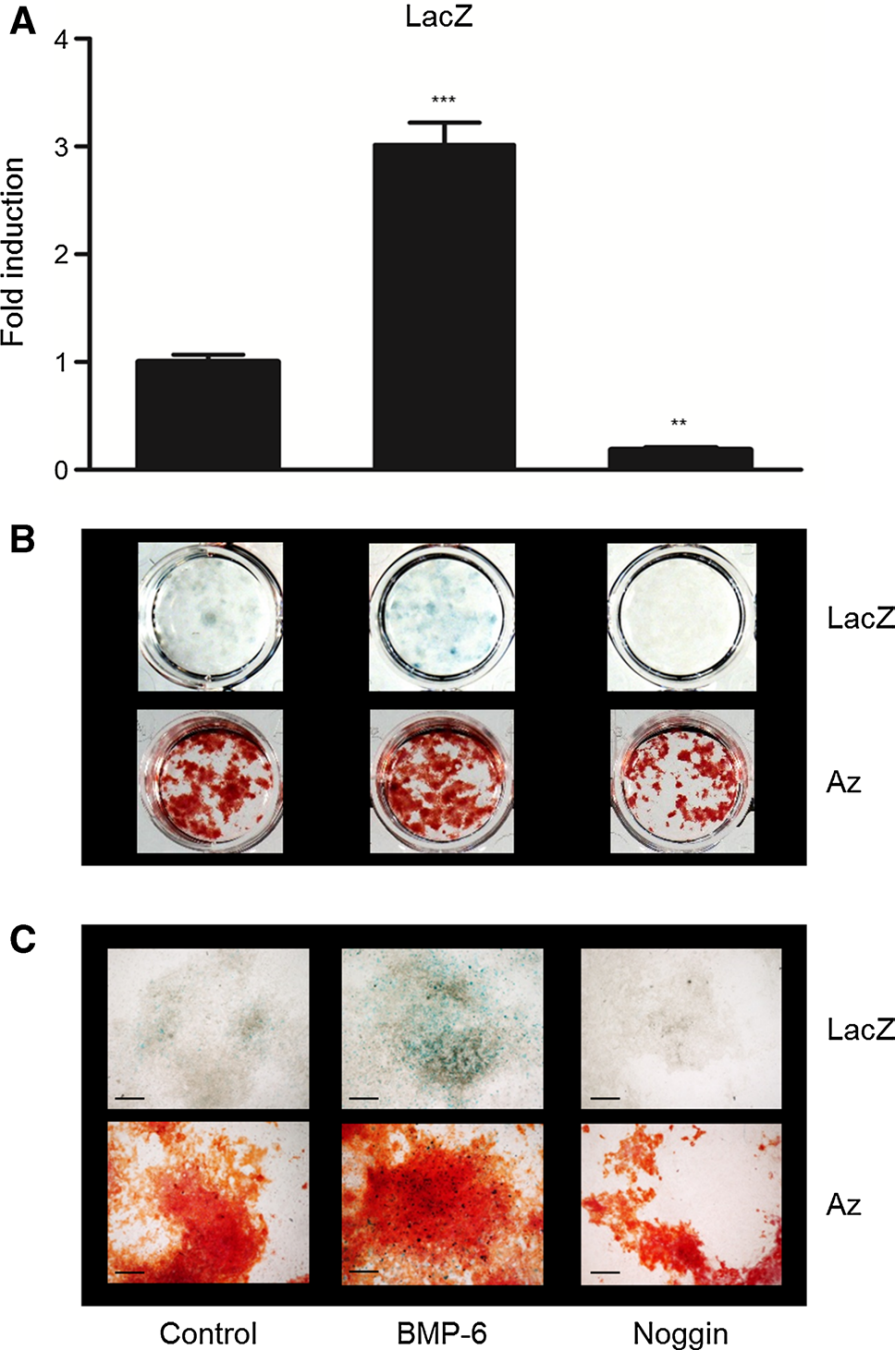
BMPs stimulate, while Noggin inhibits the differentiation of osteocytes. Bone marrow stromal cells (MCS) of RPTPμ-knock-out/LacZ knock-in mice were cultured under osteogenic conditions for 21 days. Thereafter, the cell cultures were processed for β-galactosidase activity analysis by a LacZ enzymatic assay (**a**) or X-gal staining (**b**, **c**). Mineralization of the cultures was examined by alizarin *red* S staining (**b**, **c**). The enzymatic assay shows that BMP-6 clearly stimulates, while Noggin inhibits osteocyte formation. Staining of the cultures with X-gal confirms this observation. The wells stimulated with BMP-6 show more *blue* staining than the control wells; the wells cultured in the presence of Noggin show hardly any *blue* staining (**b**). The same observation can be made from the larger magnification of the wells (**c**). Alizarin red S staining is also stimulated by BMP-6 and reduced by Noggin, but to a lesser extend (**b**, **c**). *Bars* 200 µm; **p < 0.01; *** *p* < 0.001

When the MSC cultures were stained for β-galactosidase activity, blue cells could be observed within the nodules. However, these cells were difficult to observe and quantify since they are embedded within the (calcified) matrix (Fig. 3). To quantify the amount of osteocytes formed within the cultures, we used an enzymatic LacZ assay, in which the yellow *o*-nitro-phenol is hydrolyzed from the substrate *o*-nitro-phenyl-β-D-galactopyranoside by β-galactosidase. MSCs were cultured for 21 days with or without BMPs or Noggin and analyzed for β-galactosidase activity. In the presence of BMP-6, a twofold to fourfold increase in β-galactosidase activity could be observed (Fig. 4a). BMP-4 also increased β-galactosidase activity, but to a lesser extent (1.5–2 fold). Noggin strongly inhibited the formation of bone nodules and therefore also osteocytes, which coincided with a reduction of β-galactosidase activity to approximately 20 %. Mineralization of the cultures was increased by BMPs and decreased by Noggin, as can be observed from the alizarin red S staining, though to a lesser extent (Fig. 4b, c).

### Expression of osteoblast and osteocyte markers

**Fig. 5:**
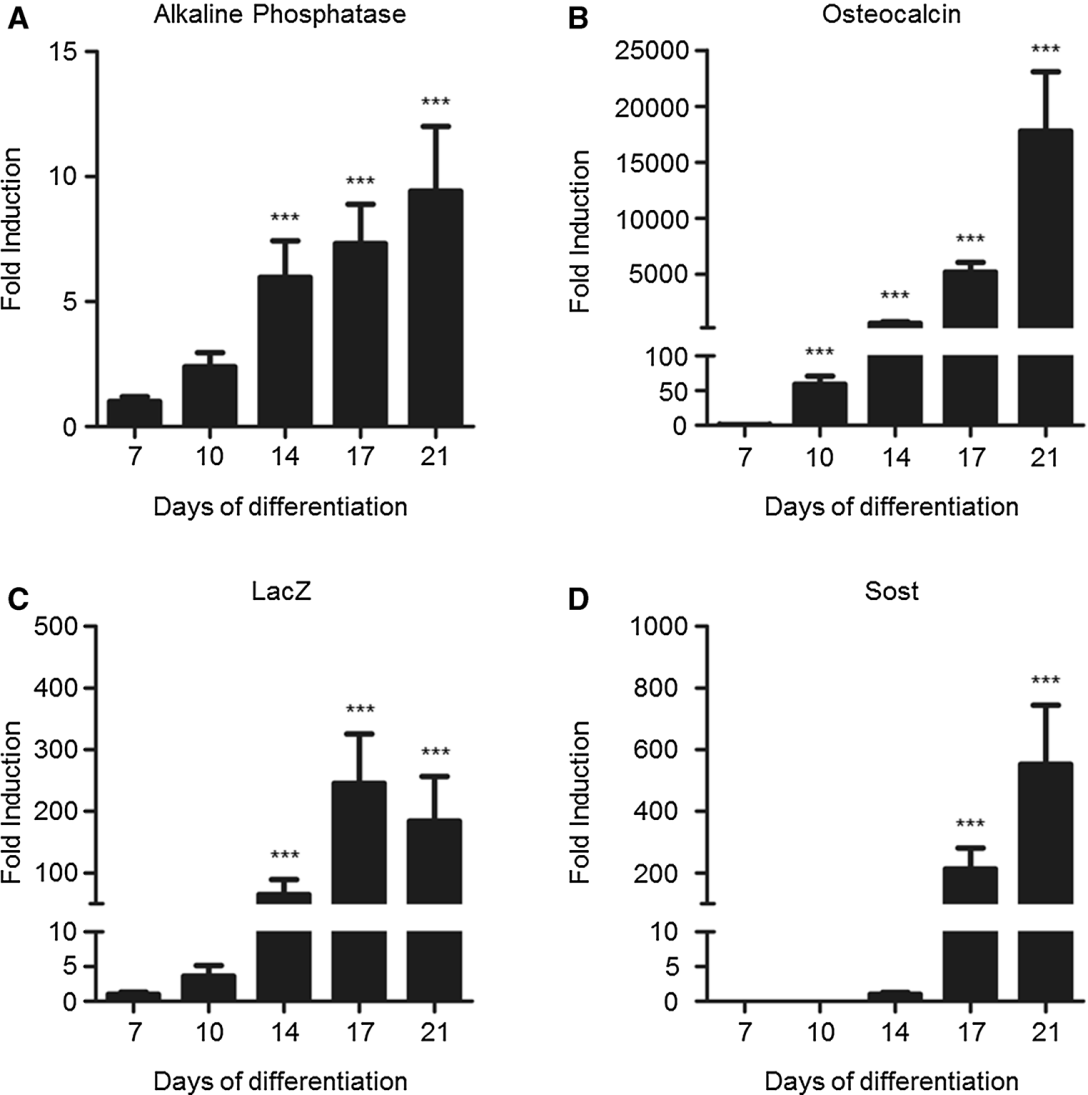
Expression of osteoblast and osteocyte markers. RNA was isolated from RPTPμ-knock-out/LacZ knock-in MSC cultures at days 7, 10, 14, 17 and 21 of differentiation. Expression of osteoblast [alkaline phosphatase (**a**) and osteocalcin (**b**)] and osteocyte [LacZ (**c**) and Sost (**d**)] markers was analyzed by quantitative RT-PCR with the housekeeping gene β2-microglobulin as internal control. Measurements were performed in triplo, and results are shown as expression relative to day 7 (**a**–**c**) or day 14 (**d**), the first day that expression could be shown, calculated using the 2^−ΔΔCt^ method. ***p < 0.001 compared with day 7 (**a**–**c**) or day 14 (**d**)

The expression of osteoblast (alkaline phosphatase, osteocalcin) and osteocyte (LacZ, Sost) markers in the MSC cultures over time was analyzed by quantitative RT-PCR. Expression was normalized to the expression of the housekeeping gene β2-microglobulin, and relative expression to day 7 (day 14 for Sost) was calculated using the 2^−ΔΔCt^ method. Within the MCS cultures, alkaline phosphatase (Fig. 5a), osteocalcin (Fig. 5b), LacZ (Fig. 5c) and Sost (Fig. 5d) expression increased during differentiation. Alkaline phosphatase, osteocalcin and LacZ are present from day 7; sclerostin is expressed from day 14 onwards. While LacZ reached a maximum expression at day 17, Sost expression was the highest at day 21. Since LacZ expression is restricted to osteocytes as is the *Sost* gene, the expression of LacZ should coincide with the expression of Sost. However, these results indicate that RPTPμ expression, represented by LacZ in these MSCs, occurs earlier in osteocyte development than sclerostin.

### Effects of RPTPµ deficiency on bone micro-architecture at several stages of development

We performed ex vivo micro-CT on the left tibiae of female RPTPμ-knock-out/LacZ knock-in mice and their WT littermates at the age of 8, 20, 32, 44 and 56 weeks to determine the effect of RPTPµ deficiency on both cortical and trabecular bone micro-architecture. At sacrifice, total body weight was determined and showed no significant differences between WT and RPTPμ-knock-out/LacZ knock-in mice (data not shown). No significant differences in bone micro-architecture between WT and RPTPμ-knock-out/LacZ knock-in mice were observed at any age except at 20 weeks.

**Fig. 6:**
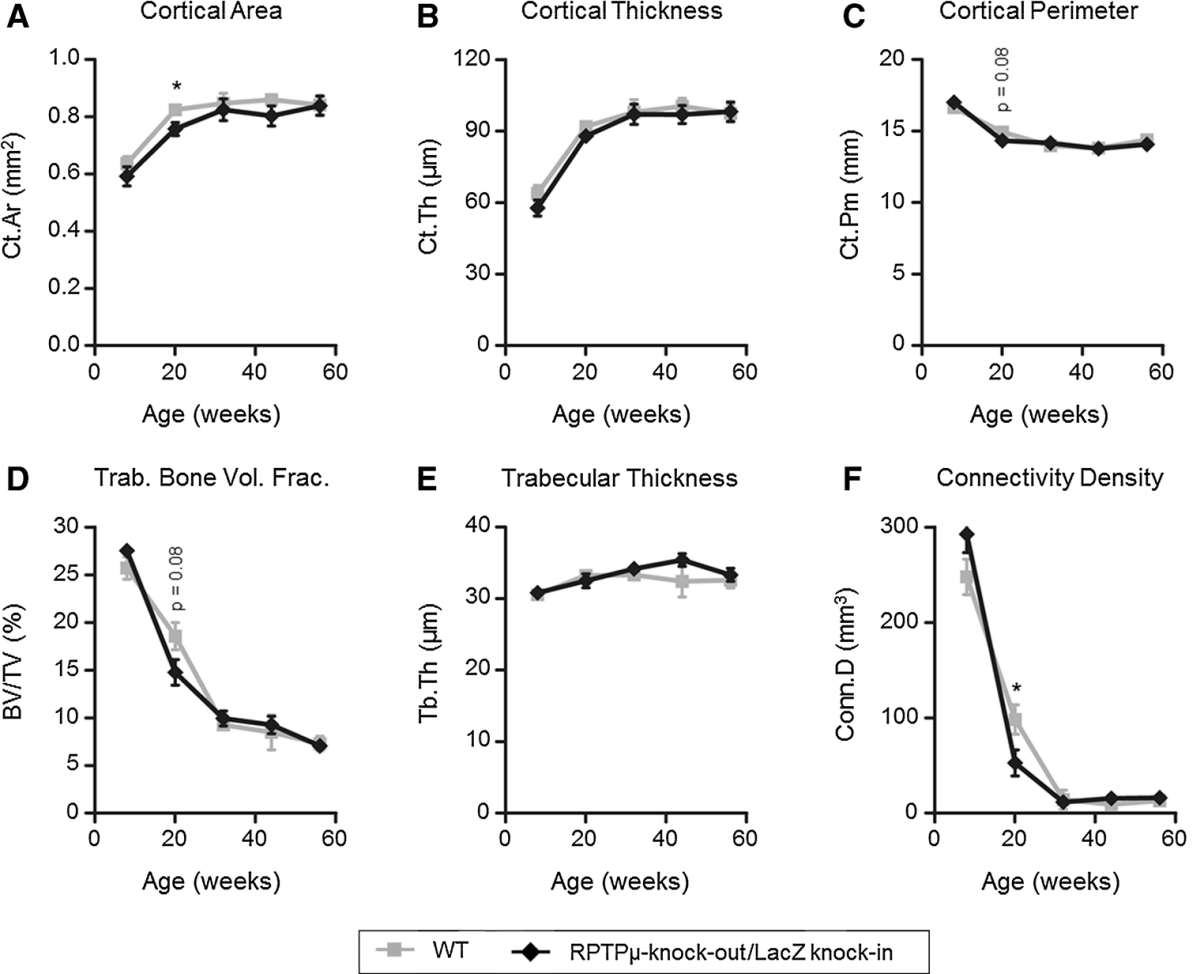
Analysis of bone micro-architecture by ex vivo µCT. Bone micro-architecture of RPTPμ-knock-out/LacZ knock-in mice and wild-type FVB mice was analyzed by scanning tibiae of 8-, 20-, 32-, 44- and 56-week-old mice (*n* = 5–9 per genotype/age group) with a SkyScan 1076 X-ray microtomograph. RPTPμ-knock-out/LacZ knock-in mice show a significantly lower cortical area (**a**) at 20 weeks in the proximal tibia compared with wild-type mice. Cortical thickness (**b**) was unaltered and cortical perimeter (**c**) was slightly lower in RPTPμ-knock-out/LacZ knock-in mice, although this difference was not significant (*p* = 0.08). RPTPμ-knock-out/LacZ knock-in mice show a slightly lower trabecular bone volume at 20 weeks (**d**), although this difference was not significant (*p* = 0.08). Trabecular thickness (**e**) was unaltered and trabecular connectivity density (**f**) was significantly lower in RPTPμ-knock-out/LacZ knock-in mice. *p < 0.05

Cortical bone mass was lower in 20-week-old RPTPμ-knock-out/LacZ knock-in mice as exemplified by lower cortical area (*p* < 0.05 vs. WT, Fig. 6a) and cortical volume (0.374 ± 0.011 mm^3^ vs. 0.407 ± 0.006 mm^3^, *p* < 0.05 vs. WT). Figure 6 also shows cortical thickness (b) and cortical perimeter (c), which had a tendency to be lower in RPTPμ-knock-out/LacZ knock-in mice, but these differences did not reach statistical significance (*p* = 0.14 and *p* = 0.08 vs. WT, respectively). Altogether these results show that tibiae of RPTPμ-knock-out/LacZ knock-in mice did not significantly differ in size from tibiae of WT mice, but did contain less cortical bone, suggesting that they have lower bone strength. This was supported by a significantly lower mean polar moment of inertia, which is a proxy for cortical bone strength (0.558 ± 0.030 mm^4^ vs. 0.644 ± 0.015 mm^4^, *p* < 0.05 vs. WT).

Besides these clear effects on cortical bone at 20 weeks, we also detected more subtle changes in trabecular bone of RPTPμ-knock-out/LacZ knock-in mice compared with WT mice at this age. Trabecular bone volume fraction tended to be lower in RPTPμ-knock-out/LacZ knock-in mice (*p* = 0.08 vs. WT, Fig. 6d), which is certainly not caused by changes in trabecular thickness (*p* = 0.53, Fig. 6e), but more likely by decreased connectivity density (*p* < 0.05 vs. WT, Fig. 6f), indicating a lower degree of interconnection within the trabecular network. This may be explained by a combination of factors, namely less trabeculae that appear more rod-like in shape and are further shaped apart, as shown by trends toward lower trabecular number (1.54 ± 0.14 mm^−1^ vs. 1.88 ± 0.15, *p* = 0.14 vs. WT), increased structure model index (0.129 ± 0.016 mm^−1^ vs. 1.876 ± 0.146 mm^−1^, *p* = 0.08 vs. WT) and increased trabecular separation (166 ± 10 µm vs. 141 ± 11 µm, *p* = 0.11 vs. WT).

## Discussion

In the present study, we show that within bone tissue, RPTPµ is exclusively expressed in osteocytes and that osteocytes are formed within the bone nodules of MSC cultures, which can be visualized by light microscopy and quantified by an enzymatic assay. Staining of osteocytes for β-galactosidase activity illustrates that early-stage osteocytes express LacZ, in contrast to the osteocyte-specific protein sclerostin, which is only present in mineralized osteocytes (Poole et al. 2005). Sclerostin, the protein of the Sost gene, is an osteocyte-specific inhibitor of bone formation (van Bezooijen et al. 2004), which when absent results in the sclerosing bone disorder sclerosteosis (Balemans et al. 2001; Brunkow et al. 2001). The Sost gene is expressed from day 14 in MSC cultures and from day 11 in the murine osteoprogenitor cell line KS483 (van Bezooijen et al. 2004). DMP-1 is an extracellular matrix protein present in osteocytes in adults (Toyosawa et al. 2001). Kalajzic et al. (2004) have shown that in MSC cultures of transgenic mice expressing the green fluorescent protein (GFP) under control of the DMP-1 promoter, within the nodules osteocytes are present from day 13 onwards. Our data on LacZ expression concur with these results. However, since in RPTPμ-knock-out/LacZ knock-in mice the LacZ gene is expressed by knock-in, the formation of osteocytes within cell cultures can be quantified. This provides an excellent tool for the study of compounds on osteocyte differentiation in vitro.

Interestingly, membrane-associated tyrosine phosphatases have been identified in rat osteosarcoma cells (Southey et al. 1995; Lezcano et al. 2014).

RPTPµ plays a role in cell–cell interaction and therefore is expressed in cell types in which this interaction is very important, e.g., osteocytes, endothelium, nerve cells and cardiac muscle cells. Within endothelium, expression varies and is increased with increasing cell–cell interactions (Koop et al. 2003). In fenestrated endothelium, which is present in the liver, spleen and bone marrow, expression of RPTPµ is very low (Bianchi et al. 1999). In the brain, RPTPμ has been shown to be involved in axon guidance and neurite outgrowth (reviewed by Johnson and Van Vactor (2003); Ensslen-Craig and Brady-Kalnay (2004)). We have shown that RPTPμ is expressed in early osteocytes. These osteoid osteocytes are developing the cellular processes which form the cellular network. RPTPμ might be involved in the process, although the absence of RPTPμ does not prevent the processes from developing.

Osteocytes form a three-dimensional network within the mineralized matrix of the bone. This network is important for the supply of oxygen and nourishment to the osteocytes. However, cell–cell interactions may also play a role in the signaling of mechanical loading of osteocytes to the cells on the surface of the bone. In RPTPµ-deficient mice, Koop et al. (2005) have shown that RPTPµ is involved in the mechanotransduction or accessory signaling pathway that controls shear stress responses in mesenteric resistance arteries. It has been shown that fluid flow shear stress plays an important role in osteocyte mechanotransduction (Klein-Nulend et al. 1995; Fritton and Weinbaum 2009; Price et al. 2011); therefore, RPTPµ might have a similar function in osteocytes.

Giepmans et al. (2003) showed that RPTPµ can bind directly to connexin-43, while Lezcano et al. (2014) have shown that bisphosphonates can decrease this association in ROS17/2.8 osteosarcoma-derived osteoblasts. Connexin-43 is present in osteocytes, where they form gap junctions and hemichannels. Connexin-43 hemichannels are mechanosensory in nature as they open when subjected to fluid flow shear stress, resulting in the magnitude-dependent release of PGE2 that is involved in bone remodeling (Siller-Jackson et al. 2008). The absence of connexin-43 in osteoblasts/osteocytes protected against unloading, while increasing the effect of mechanical loading (Lloyd et al. 2013; Bivi et al. 2013).

Since connexin-43 hemichannels seem to play a crucial role in fluid flow shear stress-induced mechanotransduction, plus the fact that RPTPµ can directly bind to these hemichannels, it is tempting to suggest that RPTPµ is also involved in osteocyte mechanotransduction. Further studies are needed to investigate this.

Osteocytes depend on cell–cell interactions with other osteocytes and osteoblasts on the surface of the bone for nutrients and oxygen. In cortical bone, it is likely that cell–cell interactions play a larger role than in trabecular bone, since the distances to the surface of the bone are larger in osteons in cortical bone than in trabecular bone. This might explain why we have observed a clear reduction in cortical bone area and cortical volume using μCT analysis and more subtle differences in trabecular bone, becoming apparent at the age of 20 weeks. However, later in development the differences disappeared, suggesting that RPTPµ plays a major role around the age of 20 weeks, an important period in the development of the skeleton. These results were confirmed by BMD analysis using a PIXImus densitometer, which showed a lower, not significant, BMD in the femur of RPTPμ-knock-out/LacZ knock-in mice compared with WT littermates, but no differences in the lumbar spine (data not shown). Although we have observed these differences, no other apparent bone phenotype is present in the RPTPμ-knock-out/LacZ knock-in mice. Since RPTPμ belongs to a large family of proteins, the effect of deletion of one of its members may be compensated by another family member.

Future research of the histological and biological properties of bone of RPTPμ-knock-out/LacZ knock-in mice might provide more information on the importance of this gene in osteocytes. Information about osteocyte function might provide therapeutic tools for intervention in diseases in which the balance between bone formation and bone resorption is disturbed, e.g., osteoporosis. In addition, RPTPµ itself may be a target for therapy in bone disorders.
